# New Codeism in Alabama

**Published:** 1888-04

**Authors:** J. D. S. Davis

**Affiliations:** Birmingham, Ala.


### NEW CODEISM IN ALABAMA.

*Editors Atlanta Medical and Surgical Journal:*

The State Medical Association of Alabama has taken a new departure, and now by legislation favors the new code of ethics, while at the same time there remains a provision in its constitution claiming to foster and practice the code of ethics of the American Medical Association.

For a long time in the history of Alabama two classes of practitioners of medicine, regular and irregular, were recognized. But within the past two years the law to regulate the admission of applicants to practice medicine in the State of Alabama has been so amended and materially changed that the words regular and irregular have been made to disappear from its provisions.

The monument to the honor of Alabama doctors, the Medical Laws of Alabama, for a long time contained certain restrictions which often sorely tried those physicians, "irregular," known as eclectics and homœopathics. Fretting under the close-fitting harness, so adroitly adjusted by the Medical Association of Alabama, the eclectics began a continuous kick, and their attempt to influence medical legislation was met by the State Medical Association of Alabama with such a radical change of the provisions of the law as to meet every demand of this school of irregular practitioners of medicine*. The State Medical Association of Alabama realized that the passage of any such law contemplated by the Eclectic Association of Alabama would pave the way to admit to practice in the State all the quacks and ignoramuses who chose to flock into Alabama ; that ignorant graduates, rejected by the regular boards of examiners, could apply to this new board and obtain certificates to practice the reform or the eclectic system of medicine ; and, too, that the law of the State could and would be so revised as to make the distinction among doctors more emphatic, and

*See Transactions of the State Medical Association of Alabama for 1880 and 1881; also Transactions State Medical Association of Alabama for 1885, pp. 136-139.

the law was so amended by the State Medical Association as to revise the requirements or standard of qualification to be required of the applicants for the several State and county boards of examiners as to meet the wish of eclectics and homœopaths and to conform to public opinion generally, creating thereby such a complete reformation or change in the medical affairs of the State as to make all who receive a diploma or certificate to practice medicine in the State of Alabama, though irregular or defective, honorable, regular doctors.

The law now provides that all applicants, from whatever school, to practice medicine in the State shall stand an examination in the following branches of medicine: Science of chemistry, anatomy, physiology, operative surgery and surgical anatomy, mechanicism of labor and operative midwifery, natural history of diseases, including pathology, physical diagnosis, medical jurisprudence, public and private hygiene, practice and materia medica being left out of the schedule of examination that there might be no distinctive difference or diversity of doctrines possible. And though applicants, graduates of antagonistic schools, should stand examinations before the same board, there is no distinctive difference when diplomas are granted by the board authorized to make such examinations by the State Medical Association of Alabama.

Now, since the real barrier between the different schools of medicine is destroyed by admission to the profession of medicine regularly through the requirements of the existing medical laws of Alabama, it no longer remains a question of practice, but a question of ethics.

If this be true, and in accordance with the principles of justice, the representatives of all schools being made regular through the requirements of the medical laws of the State, all stand equal before the law, the people and the profession. Hence each licentiate is entitled to "individual exercise and honors of the profession." The words regular, irregular and pathies of every kind no longer exist in the provisions of the law. Every doctor gets the same kind of a certificate or diploma, is examined by the same boards, authorized and recognized by the State Association.

The State Medical Association claims to practice the ethics of the American Medical Association (its constitution providing for it). But if, by accepting eclectics and homœopathics into the profession by legislation, they are made legally, morally and socially equal with the members constituting the State Association, they are relatively equal professionally. If the schedule of examination laid down affords evidence of professional proficiency, so long as any (heretofore irregular) individual doctor does not practice medicine to the exclusion of the knowledge to be derived from the several branches examined in, is entitled by legislation to recognition by the profession and "individual exercise and honors of the profession" of the State of Alabama. And this is in conformity with the teachings of the code of ethics of the American Medical Association. Art. IV.., section I, of that code reads: "A regular medical education furnishes the only presumptive evidence of professional ability and ought to be the only acknowledged right of an individual to the exercise and honors of this profession." The State Association, to be consistent with the teachings of its own constitutional government, cannot exclude from membership its own *regular alumni*, or prevent its members from consulting with the homœopath and eclectic, where such a consultation is desired by the patient. If, then, as every intelligent thinker can but see, this state of affairs exists, the new code is the only prevailing ethics of the State Association. Many members of this Association, whose ethical characters are above reproach, have unconsciously fallen victims to this glaring error of the Association.

The modification of the medical examination was made to harmonize an irregular faction, and if it does not work harm to the profession of Alabama, it cannot fail to place the State Association in harsh opposition to the mother of American medical organization, the American Medical Association.

The State Association fearing that adverse legislation would be had for the protection of homœopathy and eclecticism, a modification was influenced with a hope of meeting their wants and requirements. In the change the regular medical profession of Alabama is allied by legislation with the homœopathic and eclectic professions.

Since (in Alabama) a thorough knowledge of chemistry, anatomy, physiology, operative surgery and surgical anatomy, mechanism of labor and operative midwifery, natural history of diseases, including pathology, symptomatology, physical diagnosis, public and private hygiene, is *presumptive evidence of professional ability*. Whether these branches be found taught in the curriculum of a regular homœopathic or eclectic school, if the individual who has been found proficient in all is of good moral character, and does not practice some exclusive dogma to the rejection of the aids actually furnished by these several branches of medicine, members of the State Medical Association of Alabama, in the sight of said Association, are excusable in allowing and granting homœopathic and eclectic physicians all the courtesies and privileges that would be expected of brother members of the association, or expected of any brother alumnus of the institution from which said member received a diploma entitling him to examination by a board in Alabama.

Since no pathy is recognized by the State Association, and all Alabama doctors are alumni of the same institution, viz., the State Medical Association of Alabama, the members of said institution are as excusable for receiving the aid, in consultation, of the homœopathic surgeon, gynæcologist, obstetrician, and diagnostician, as the professors of any given medical college would be in receiving a like service from one of its alumni.

My friend, Dr. T. D. Park, in a paper read before the Jefferson County Medical Society, in February, 1888, suggested by the fact that all the physicians of his acquaintance who had expressed themselves held ethical views so opposed to those as forced on him as a resort of a study of the ethics of the State Association of Alabama, said: "The State and county boards of the Alabama State Association, composed of the censors of the respective State and county societies, have *sole* legal power to issue license to practice—*i. e.*, diplomas in the State. Diplomas or certificates from no college, university or medical board are recognized. All applicants come on equal footing and are subject to the same examination. . . . As the good of the patient is the sole object in view, no intelligent practitioner, who has a license to practice from some board of known respectabilty, and who is in good standing in the place where he resides, should be excluded from fellowship, or his aid refused in consultation. But no one can be considered as a regular practitioner whose practice is based on exclusive dogma to the rejection of the accumulated experience of the profession and the aid furnished by anatomy, physiology, etc., etc.

"The certificate—the diploma of a board—furnishes in the State of Alabama the only possible evidence of a regular and sufficient medical education. Since no other diploma or certificate is recognized as furnishing this evidence, consequently every person who holds one of these certificates holds in it the proof of a regular medical education, and hence the right to the exercise and honors of his profession. Since the Alabama boards are of known and acknowledged respectability, it follows that every licensed physician in the State, whose conduct conforms to that of a physician and gentleman, may not be excluded from fellowship—*i. e.*, membership. It were too strong a reflection upon the integrity of any Alabama board to assert that it would license candidates to practice upon the people of the State who, upon examination, found by their ignorance that they rejected the accumulated experience of the profession or the aid of anatomy, physiology, etc. Moreover, no homœopathic claims to reject the accumulated experience of the profession. He only claims the right to reject such parts of it as agree with his opinion—a claim held by every thinking physician."

Dr. Park again said: "Unlike the New York State Medical Association, the State Association of Alabama, in order to take an advanced (?) and liberal stand on ethics, did not antagonize the American Medical Association, but simply induced the legislature of the State to pass laws that placed all licensed physicians on an equal plane, that make all legal practitioners regular, and hence it is enabled to permit its members, in due conformity with the national code, to meet physicians in good moral and professional standing, who, in other States, might be known as homœopaths, thus proving the truth of one expression heard by me several times, but not understood in its entirety, "that the medical laws of Alabama are in advance of those of any other State in the Union."

"And manifestly, the Alabama Association cannot refuse fellowship to any licensed physician in good standing in the State from the fact that it is the only *alma mater* that any Alabama physician can legally claim; and the Association cannot so stultify itself as to declare that certain of its *alumni* are not competent, even on the day of their graduation, to be received into fellowship or met in consultation." "What would be thought of an army examining board that would recommend an applicant for appointment and then, in the face of this recommendation, take the position that the appointee was unfit for association with the other surgeons of the army? Than the army board, no more can the State Medical Association of Alabama—trusted guardian of their interest—declare to the people of the State that it licenses incompetent, unfit, unprincipled men to ply their nefarious trade among them."

Alabama is the pioneer State in medical organizations and has encountered no obstacles it has not overcome, and it is hoped that present and future barriers may yield to its demands.

The Association is composed of that alchemy of human purpose and genius which has touched the crude elements of an imperfect organization and converted them into progress and greatness.

Yet, in a hasty chase after the mendacious bubble, it has exhibited a degree of recklessness if not of folly. An association which is stamped with such persistency of effort and human endeavor as to surpass all others in far-reaching results should not have been guilty, in the face of its diametrically arranged constitution, of influencing legislation, which, from its very nature, must and has become a part of the provisions of said Association, without first considering the ultimate result.

It has soiled its good name and placed a question of ethics in the festive dish that will be refused by the very guests sought to be palliated. Like the Sioux who, after banqueting at the board of Colonel Pike, rose up and slew him ; like a coal of fire, "if it does not burn it will smart."

Unqualifiedly this was not the intent of the change, but it furnishes the result.

If the profession is not prepared to realize that this is the significance of thus allying the regular with the irregular profession, time in silent oratory will prove the truth of my statement.

I congratulate the Association on much of its work, and hope that in this last legislation it has not misused its influence and power. J. D. S. DAVIS, M. D.

*Birmingham, Ala., March 10, 1888.*

